# Monolaurin Confers a Protective Effect Against Porcine Epidemic Diarrhea Virus Infection in Piglets by Regulating the Interferon Pathway

**DOI:** 10.3389/fimmu.2021.797476

**Published:** 2022-01-13

**Authors:** Qian Zhang, Dan Yi, Changzheng Ji, Tao Wu, Manli Wang, Shuangshuang Guo, Lei Wang, Di Zhao, Yongqing Hou

**Affiliations:** Hubei Key Laboratory of Animal Nutrition and Feed Science, Wuhan Polytechnic University, Wuhan, China

**Keywords:** monolaurin, porcine epidemic diarrhea virus, interferon, piglets, infection

## Abstract

Porcine epidemic diarrhea virus (PEDV) has reemerged as the main pathogen of piglets due to its high mutation feature. Monolaurin (ML) is a natural compound with a wide range of antibacterial and antiviral activities. However, the role of ML in PEDV infection is still unknown. This study aimed to evaluate the effect of ML on the growth performance, intestinal function, virus replication and cytokine response in piglets infected with PEDV, and to reveal the mechanism through proteomics analysis. Piglets were orally administrated with ML at a dose of 100 mg/kg·BW for 7 days before PEDV infection. Results showed that although there was no significant effect on the growth performance of piglets, ML administration alleviated the diarrhea caused by PEDV infection. ML administration promoted the recovery of intestinal villi, thereby improving intestinal function. Meanwhile, PEDV replication was significantly inhibited, and PEDV-induced expression of IL-6 and IL-8 were decreased with ML administration. Proteomics analyses showed that 38 proteins were differentially expressed between PEDV and ML+PEDV groups and were significantly enriched in the interferon-related pathways. This suggests ML could promote the restoration of homeostasis by regulating the interferon pathway. Overall, the present study demonstrated ML could confer a protective effect against PEDV infection in piglets and may be developed as a drug or feed additive to prevent and control PEDV disease.

## Introduction

Porcine epidemic diarrhea virus (PEDV) belongs to coronavirus which are enveloped, positive-sense RNA viruses. These viruses are well known for their high probability of developing mutant strains (1). Therefore, PEDV has become the main cause of piglet diarrhea throughout the world (2). PEDV is highly pathogenic in newborn pigs (almost 100% mortality), and the mortality negatively correlates with the age of the pig. Histopathologic changes were mainly observed in the small intestine, although PEDV antigen was also detected in other parts of the intestine. After PEDV infection, the intestinal epithelial cells (enterocytes) would undergo apoptosis or necrosis. Therefore, PEDV-induced diarrhea is more likely to be a consequence of malabsorption due to the massive loss of absorptive enterocytes (3). In addition to its genetic diversity, PEDV has also evolved a variety of strategies to antagonize host innate antiviral defense for successful infections (4). Until now, safe and effective drugs and feed additives are unavailable.

Monolaurin (ML), also known as glycerol monolaurate or lauroyl, is a natural compound mainly found in coconut oil and human breast milk (5). Now, it is on the list of FDA’s generally recognized as safe (GRAS) substances and available as a nutritional supplement for various products (5, 6). Monolaurin has presented antibacterial and antiviral activities in several lines of evidence (7–10). Promising results have been observed with HIV-infected rhesus macaques, suggesting that monolaurin could effectively prevent the transmission of mucosal SIV and protect against repeated high-dose SIV challenges (8, 9). *In vitro* studies have shown that monolaurin has antiviral activity against yellow fever virus, mumps virus, and zika virus (10). These effects are achieved by dissolving the lipids and phospholipids in the pathogen’s envelope (10). Projan et al. reported that the antimicrobial effect is associated with its interference with signal transduction in cell replication (11). ML could also inhibit immune activation and the production of chemokines and cytokines in human vaginal epithelial cells (HVECs) challenged with staphylococcal toxins (12). Most importantly, it would not produce antiviral or antibacterial resistance, which makes it a promising feed additive (13). However, the effect of monolaurin on PEDV infection has not been determined.

Therefore, the purpose of this study was to evaluate the effect of ML on PEDV infection *in vivo* and determine the mechanism using proteomics analysis. These findings are expected to provide insights for the application of ML in anti-PEDV infection.

## Material and Methods

### Animal Experiments

A 2 × 2 factorial randomized complete block design was employed to study the effects of monolaurin administration (with or without oral administration of monolaurin at a dose of 100 mg/kg·BW), virus infection (with or without PEDV infection at the dose of 1×10^4.5^ TCID_50/_piglet), and their interactions. Briefly, 32 seven-day-old piglets with similar body weight were randomly assigned to one of four groups: Control group, ML group, PEDV group, ML+PEDV group. Each group was individually housed with strict control of cross infection and suitable ambient temperature and humidity. Piglets were acclimatized for three days before the start of the animal experiment. On day 4, piglets in the ML and ML+PEDV groups were orally administrated with ML (dissolved in liquid milk replacer) for 7 days. The Control and PEDV groups were administrated with the same volume of liquid milk replacer. On day 8, piglets in the PEDV and ML+PEDV groups were orally administrated with PEDV PBS solution, and the other two groups were received the same volume of PBS solution. On day 11, all piglets were slaughtered under anesthesia. Health indicators, such as body weight, food intakes, diarrhea incidence, were recorded during the entire experiment. The severity of diarrhea was scored as described previously (14). Blood and intestinal samples were collected and subjected to biochemical analysis or stored at -80°C until further analysis. All procedures were approved by the Animal Care and Use Committee of Wuhan Polytechnic University.

### Intestinal Morphology

1-cm-long small intestine samples were fixed in 4% paraformaldehyde. Then, the fixed samples were dehydrated and embedded in paraffin. Sections of 6-µm thickness were deparaffinized in xylene and stained with hematoxylin and eosin (H&E). Images were obtained using a DM3000 microscope (Leica Microsystems, Wetzlar, Germany). The villus height (VH), crypt depth (CD), villus height/crypt depth (VH/CD) and villus surface area of the small intestine were measured with an Olympus BX41 microscope (Olympus, Tokyo, Japan) and Image-Pro Plus 6.0 software (Media Cybernetics, Rockville, MD) as described by Frankel et al. (15). A total of 10 intact, well-oriented crypt–villus units were measured in triplicate per section.

### Detection of Viral Loads by Real-Time Quantitative PCR

Total RNA from the small intestine were extracted using RNAiso Plus (Takara, Dalian, China) reagent. Then cDNA was synthesized using PrimeScript^®^RT reagent kit with gDNA Eraser (Takara, Dalian, China). Real-time quantitative PCR was performed using SYBR^®^ Premix Ex Taq™ (Tli RNaseHPlus) (Takara, Dalian, China). The relative viral load levels were evaluated by detection of N gene expression of PEDV. Gene expression was determined using the 2^-ΔΔCt^ method relative to the values in Control group after normalization to housekeeping genes RPL4. The primer sequences used for this study were listed below. PEDV-N-F: 5’-CGCAAAGACTGAACCCACTAACTT-3’, PEDV-N-R: 5’-TTGCCTCTGTTGTTACTCGGGGAT-3’; RPL4-F: 5’-GGAAACCGTCGCGAGA-3’, RPL4-R: 5’-GCCCCAGAGACAGTT-3’.

### Detection of Cytokines by Enzyme-Linked Immunosorbent Assay (ELISA)

The levels of IL-1β, IL-6, IL-8, TNF-α in the serum were determined using the ELISA kit (RD Systems, Quantikine, USA) according to the manufacturer’s instructions.

### Protein Extraction, Digestion and LC-MS/MS Analysis

The label-free proteomic analysis was performed as previously described (16). Briefly, 150 mg jejunum tissue were homogenized in T-PER lysis buffer containing protease inhibitors. The protein in the supernatant were obtained by centrifuge and quantified by the bicinchoninic acid (BCA) assay. Next, 400 μg proteins were digested to generate peptides using filter-aided sample preparation (FASP) method. The final peptides obtained by desalination and quantification were further subjected to LC-MS/MS analysis, which was performed on the Q Exactive mass spectrometer (Thermo Fisher Scientific, USA), coupled with the Easy-Nano Ultimate 3000 UPLC system (Dionex, Thermo Fisher Scientific, USA).

### Protein Identification and Data Analysis

MaxQuant software was employed to process all the raw MS/MS spectra using the *Sus scrofa* database (22,191 sequences) downloaded from UniProtKB database on the November 10, 2018. The parameters were set as described previously (16). False discovery rate (FDR) was set to 0.01. Minimum of 7 amino acids for each peptide and minimum of 2 peptides for each protein were required for reliable identification and quantification. Proteins with >1.5-fold change or < 0.67- fold change between two samples and FDR-adjusted p ≤ 0.05 were considered to be significant differentially expressed proteins (DEPs). DEPs lists were further processed with Gene Ontology (http://www.geneontology.org/, GO) and Reactome database (http://www.reactome.org) for GO term and pathway enrichment analysis. Cluster analysis was performed by MetaboAnalyst 5.0 (https://www.metaboanalyst.ca/faces/home.xhtml). The relationships among these DEPs were depicted in Venn diagrams (http://bioinformatics.psb.ugent.be/webtools/Venn/).

### Western Blot Analysis

Tissues were homogenized in lysis buffer containing protease inhibitors and centrifuged to collect supernatants. Equal amounts of protein were separated in SDS-PAGE gels, followed by transferring onto PVDF membranes (Millipore, Billerica, MA, USA). The membranes were incubated with primary antibody overnight at 4 °C, followed by incubation with secondary antibody for 2 h at room temperature. Antibodies/dilution used in this study were as follows: anti-Mx1 (ab79609, Abcam)/1:1000, anti-DDX58 (4200, Cell Signaling Technology)/1:1000, anti-ISG15 (ab233071, Abcam)/1:1000, and anti-β-Actin (PA1-46296, Invitrogen)/1:4000.

## Results

### Growth Performance

**Figure 1:**
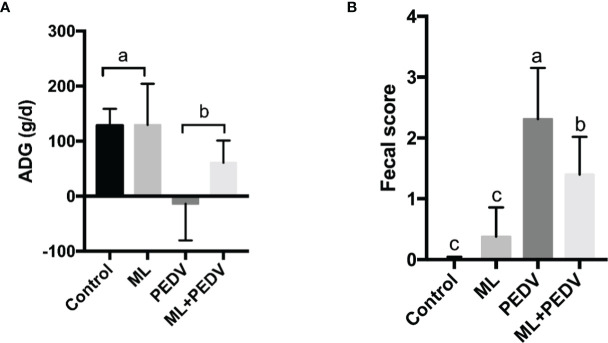
Effects of ML administration on the average daily gain **(A)** and fecal score **(B)** in piglets infected with PEDV. Bars not sharing a common lowercase letter differ significantly (p < 0.05).

Prior to PEDV infection, oral administration of ML had no effect on the growth performance, as indicated by the comparisons of average daily gain (ADG), average daily food intakes (ADFI), feed conversion ratio (FCR) and diarrhea score between +ML groups (ML group and ML+PEDV group) and no ML groups (Control group and PEDV group) (**Table 1**). PEDV infection significantly reduced ADG and increased diarrhea score compared to Control group (P<0.05) (**Figure 1**). ML administration had no effect on ADG while significantly decreased diarrhea score (P<0.05) compared to PEDV group (**Figure 1**).

**Table 1:** Effect of ML on growth performance of piglets prior to PEDV infection.

Items	-ML	+ML	*P v*alue
ADFI, g/d	88	84	—
ADG, g/d	83 ± 42	85 ± 61	0.921
FCR	1.132	1.052	—
Diarrhea scores	0.568 ± 0.617	0.715 ± 0.667	0.525

### Effect of ML on Intestinal Morphology and Function in PEDV-Infected Piglets

**Figure 2:**
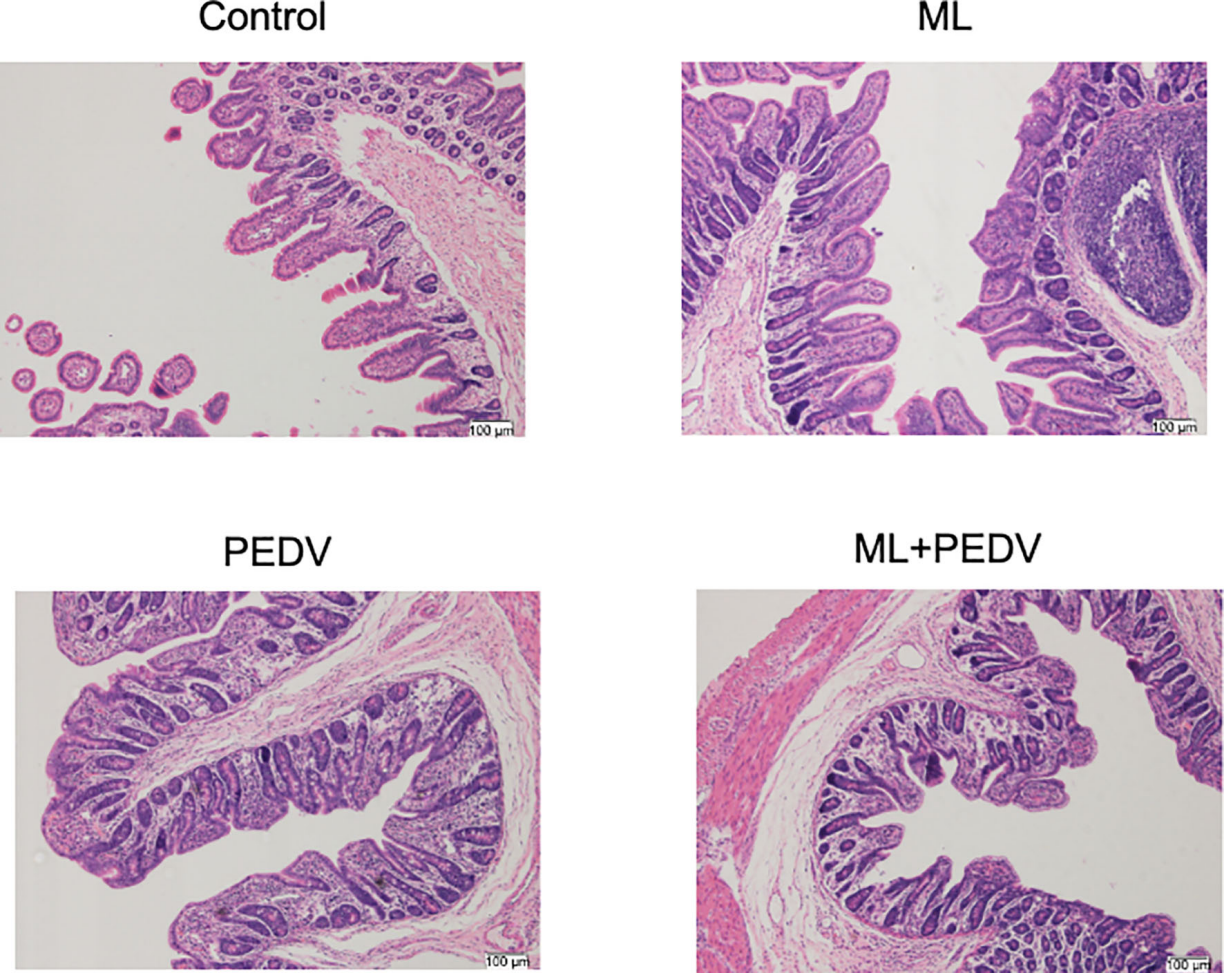
Effects of ML administration on the ileum morphology in piglets infected with PEDV. (Hematoxylin and eosin staining, ×100).

The observed damage mainly occurred in the small intestine after PEDV infection. Compared to the control group, PEDV infection significantly decreased VH, VH/CD, villus surface area, and increased CD in the jejunum. The ML administration decreased CD and increased VH/CD in the jejunum and duodenum (**Table 2**). The ileum of PEDV group was severely atrophy, and no intact villi were observed, as shown by H&E staining (**Figure 2**). The ileal villi recovered in the ML+PEDV group.

**Table 2:** Effects of ML administration on intestinal morphology in PEDV-infected piglets.

Items	-PEDV		+PEDV		SEM	*P value*		
	-ML	+ML	-ML	+ML		PEDV	ML	PEDV×ML
**Duodenum**								
Villus height (μm)	264	251	226	267	17.974	0.538	0.452	0.137
Crypt depth (μm)	69^b^	73^b^	82^a^	70^b^	3.601	0.127	0.192	0.016
Villus height/Crypt depth	4.02^a^	3.47^ab^	2.79^b^	3.92^a^	0.31	0.171	0.291	0.005
Villous surface area (μm^2^)	6965	6685	5912	6254	443	0.106	0.946	0.490
**Jejunum**								
Villus height (μm)	276	284	152	135	21.457	<0.001	0.704	0.319
Crypt depth (μm)	66^b^	65^b^	103^a^	52^b^	7.780	0.031	<0.001	<0.001
Villus height/Crypt depth	4.15	4.86	1.63	2.74	0.44	<0.001	0.007	0.526
Villous surface area (μm^2^)	6785	6462	3483	3086	546	<0.001	0.304	0.915

### Effect of ML on Viral Loads in Piglet Intestines

**Figure 3:**
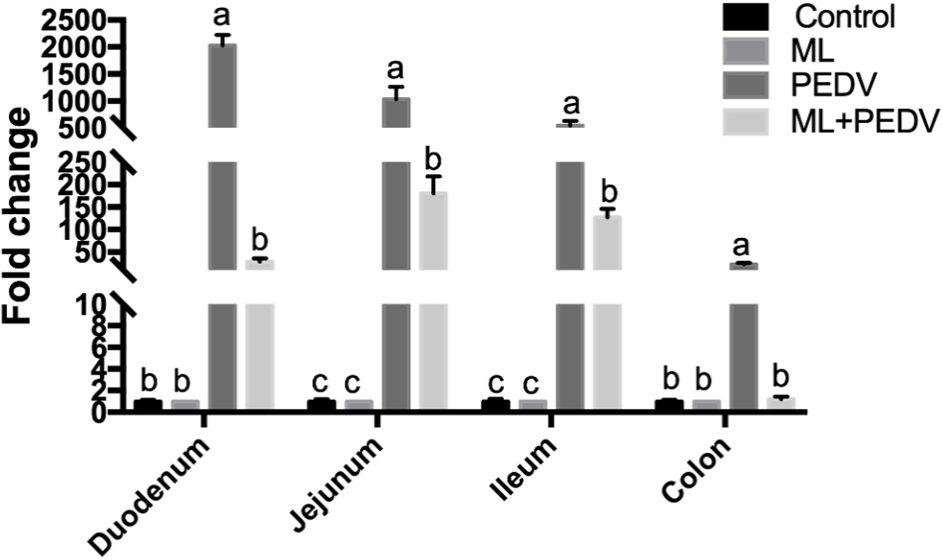
Effects of ML administration on relative viral load levels in piglets infected with PEDV. Bars not sharing a common lowercase letter differ significantly (p < 0.05).

PEDV could be detected throughout the small intestine and colon by qPCR. However, the relative viral load levels were significantly decreased in all these intestinal parts with ML administration (**Figure 3**).

### Effect of ML on Cytokine Levels in PEDV-Infected Piglets

**Figure 4:**
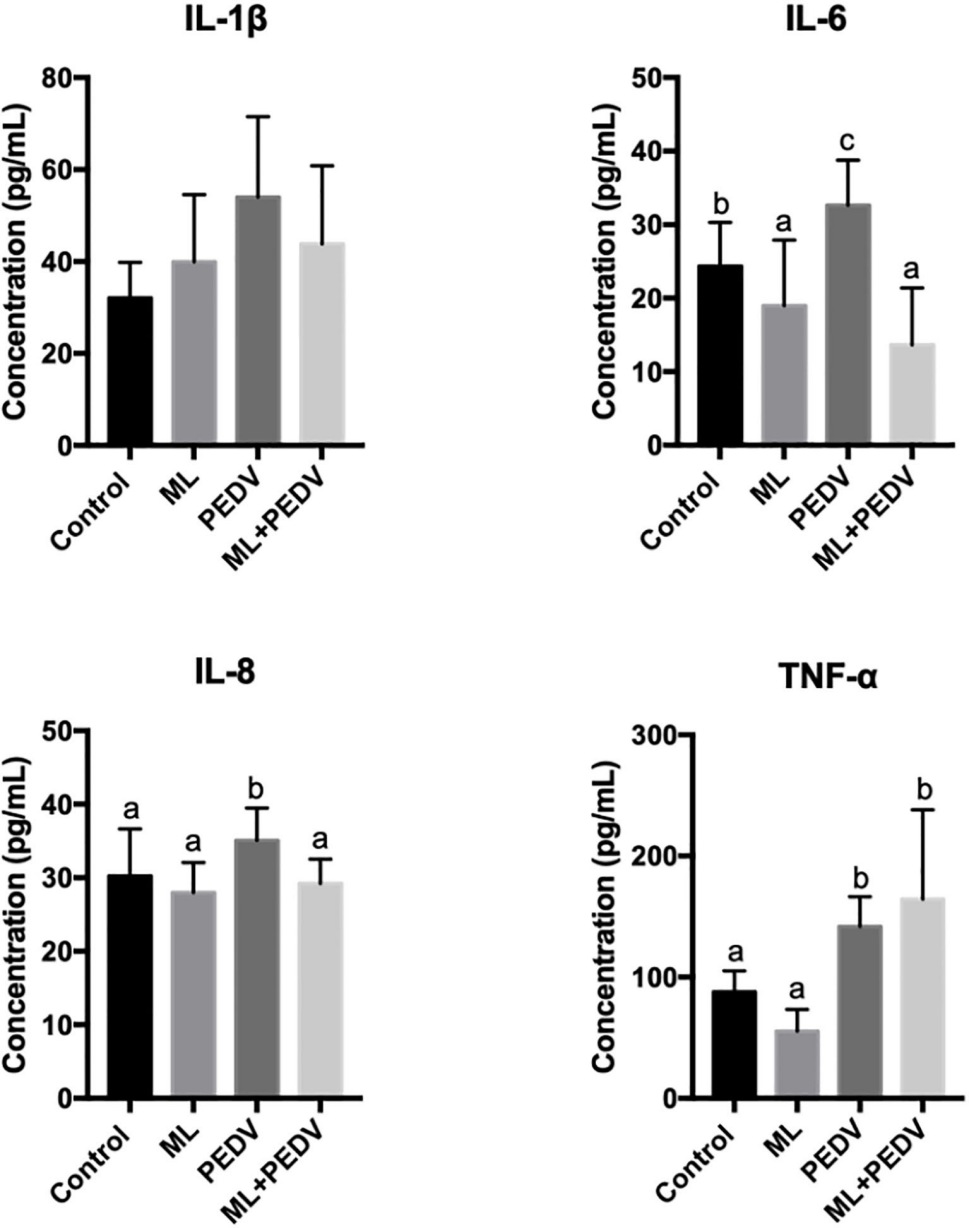
Effects of ML administration on cytokine levels in piglets infected with PEDV. Bars not sharing a common lowercase letter differ significantly (p < 0.05).

Compared with the control group, PEDV infection significantly increased serum IL-6, IL-8 and TNF-α levels. ML administration could decrease IL-6 levels in piglets with and without PEDV infection. The IL-8 level was decreased in the ML+PEDV group compared to PEDV group (**Figure 4**). There was no significant difference in IL-1β levels among these groups.

### Identification and Analysis of DEPs in the Jejunum

**Figure 5:**
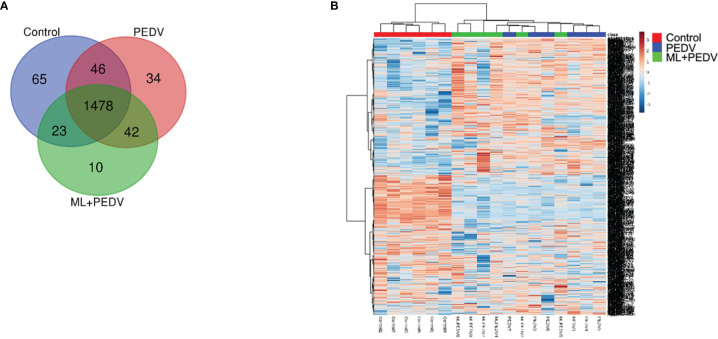
Overview of all differentially expressed proteins (DEPs) among the control, PEDV and ML + PEDV groups by proteomic analysis. The Venn diagram **(A)** showed the total number of identified proteins in the three groups. The heat map **(B)** showed the convergence of DEPs among different groups.

In order to elucidate the underlying mechanism, proteomic analysis was performed for jejunum samples in Control group, PEDV group, and ML+PEDV group. A total of 1698 proteins (corresponding to over 13536 peptides) were identified with at least two unique peptides. Out of the 1698 proteins, 1478 proteins could be found in all three groups (**Figure 5A**). The heatmap of all quantified proteins displayed distinct and specific expression patterns (**Figure 5B**).

**Figure 6:**
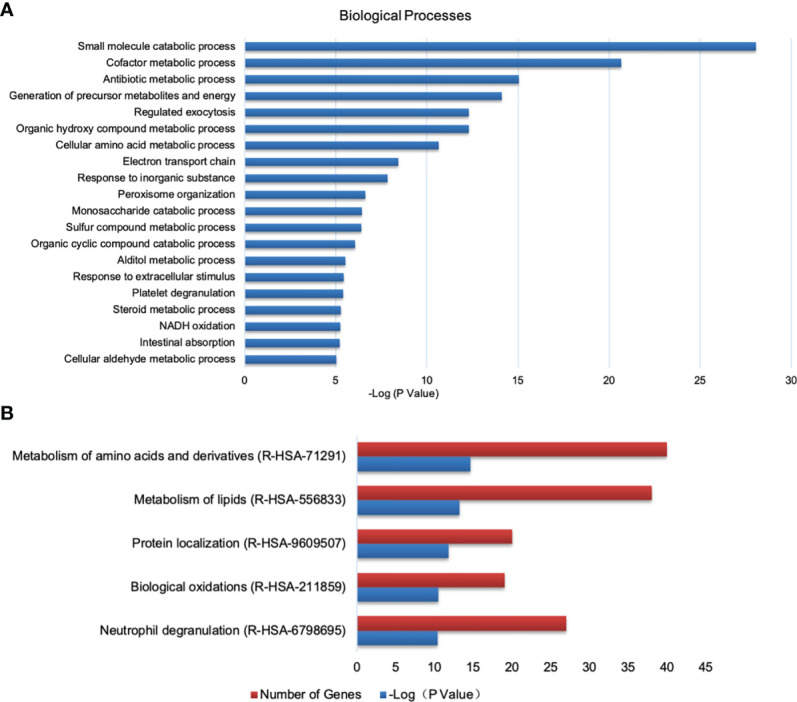
Analysis of DEPs between the control and PEDV group using GO and Reactome database. **(A)** Biological processes identified by GO enrichment. **(B)** The top 5 enriched Reactome pathways.

With the criterion of fold change >1.5 or < 0.67 and FDR < 0.05, when compared the PEDV group with the control group, 349 DEPs were identified, of which 135 proteins were upregulated and 214 proteins were downregulated. Then these DEPs were annotated at the Gene Ontology database. Results shown that proteins affected by PEDV infection were mostly involved in metabolic-related terms (**Figure 6A**). Pathway analysis shown that most DEPs were related to metabolism of amino acids and lipids, protein localization, biological oxidations, neutrophil degranulation (**Figure 6B**).

**Figure 7:**
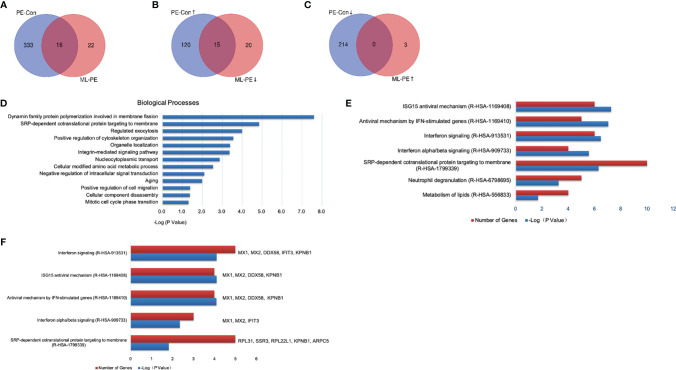
Comparison and enrichment analysis of DEPs identified among Control, PEDV and ML + PEDV groups. **(A)** The total number of DEPs in the PEDV *vs* Control (PE-Con) and ML + PEDV (ML-PE). The overlap indicated the number of common DEPs identified both in the PE-Con and ML-PE groups. **(B)** The total numbers of upregulated DEPs in the PE-Con group and downregulated DEPs in the ML-PE group. The overlap indicated the numbers of common DEPs that were upregulated in the PE-Con group and downregulated in the ML-PE group. **(C)** The total numbers of downregulated DEPs in the PE-Con group and upregulated DEPs in the ML-PE group. The overlap indicated the numbers of common DEPs that were downregulated in the PE-Con group and upregulated in the ML-PE group. **(D)** Enriched biological processes **(A, E)** the top 5 enriched Reactome pathways of the DEPs between the PEDV group and the ML + PEDV group. **(F)** Biological process enrichments for the DEPs which were reversely regulated by ML administration. ↑, upregulated; ↓, downregulated.

Compared to the PEDV group, 38 proteins were differentially expressed in the ML+PEDV group, of which three proteins were upregulated and 35 proteins were downregulated (**Figures 7A–C**). Furthermore, 15 of the 35 downregulated proteins were upregulated after PEDV infection (**Figure 7B**). After filtering with a p-value of ≤ 0.05, GO analysis of the DEPs identified between the PEDV group and ML+PEDV group showed that 13 biological processes were involved, which were presented in **Figure 7D**. Further analysis highlighted interferon related antiviral mechanism as the most significant enrichment pathways (**Figure 7E**). Coincidentally, most of the proteins that were reversed by ML administration (such as Mx1, Mx2, DDX58, IFIT3) are well-known interferon-stimulated genes (ISGs) (**Figure 7F**).

### Validation of DEPs by Western Blot

**Figure 8:**
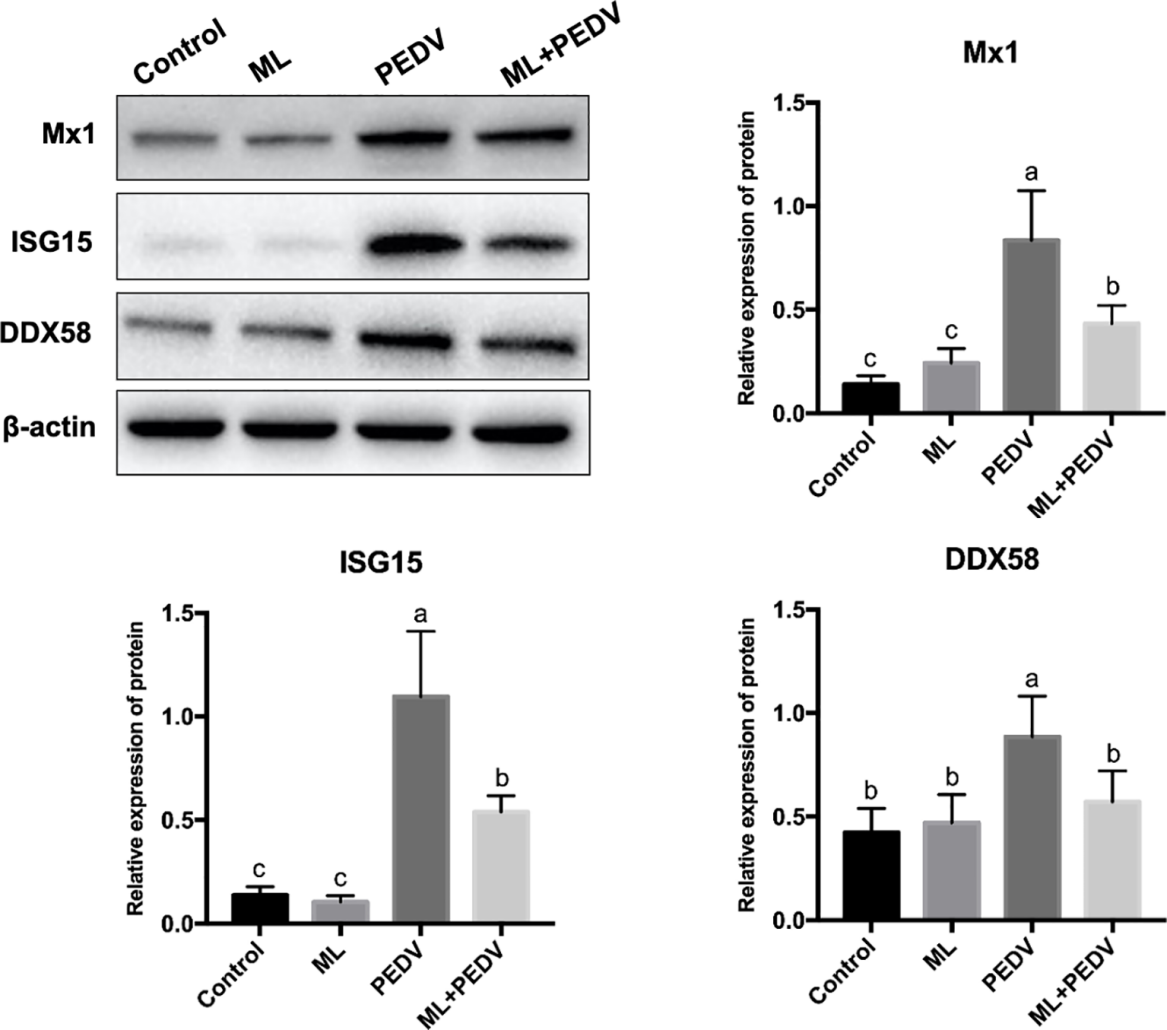
Validation of DEPs by western blot assay. Bars not sharing a common lowercase letter differ significantly (p < 0.05).

Based on the proteomic data and functional analysis, three proteins were selected for validation by western blot. The results showed that compared with the control group, PEDV infection significantly increased the abundance of Mx1, ISG15 and DDX58, which was decreased by ML administration (**Figure 8**).

## Discussion

PEDV belongs to coronavirus with high mutation properties. Therefore, the efforts to find substances with broad antiviral activity have never stopped. ML is a safe nutritional supplement. There is increasing evidence that ML has potential therapeutic effects on various viruses. However, few studies have examined the direct consequence of ML treatment on PEDV infection in piglets. To fill this knowledge gap, we examined the effect of ML treatment on PEDV infection in piglets and explored the mechanism by proteomic analysis in this study.

ML belongs to medium-chain fatty acids (MCFAs) (17). Due to its suitable taste and flavor, it is considered to be beneficial to growth performance. This view is supported by pieces of evidence in broilers (18). In the present study, ML exhibited no effect on growth performance before PEDV infection, and there were no significant differences in growth indicators between the PEDV group and the ML+PEDV group. This result is consistent with those pig studies where ML was mixed with other additives (19, 20). The discrepancy is most likely due to a species difference between broilers and pigs. Other factors may also affect the result, such as dosage, age, basal diet characteristics.

In line with our previous study, PEDV infection could induce the expression of pro-inflammatory cytokines in piglets (16). According to reports, the serum viral load of severe acute respiratory syndrome coronavirus 2 (SARS-CoV-2) is closely correlated with elevated IL-6 levels in critically ill patients (21). However, for PEDV, it is still unknown which cytokines are mainly attributable to its pathogenicity. Although ML decreased IL-6 expression level in the absence of PEDV infection, and it also decreased the expression of IL-6 and IL-8 induced by PEDV, supporting the immunoregulatory effect of ML during infection (13). Overall, the inhibitory effects on cytokine production suggest that ML may confer a protective effect during PEDV infection, as aberrant immune activation and inflammation may exacerbate the disease.

As an enterovirus, PEDV primarily infects enterocytes and causes severe damage in the small intestine (3). Our results are in accordance with this view. ML could promote the recovery of damaged villi, thereby improving intestinal function. This was supported by the lower fecal score in the ML+PEDV group. Similar results were observed by Letlole et al., who found VH: CD ratio in the duodenum, jejunum and ileum were increased with ML addition (22). The beneficial effect of ML in gut health and development may be due to the fact that ML could promote the production of energy by intestinal cells, and further promote the intestinal integrity in piglets (23).

In this study, we first demonstrated ML could inhibit PEDV replication *in vivo*. ML is active against many enveloped viruses, but not against non-enveloped viruses (10). This mechanism may be associated with destruction of the virus envelope. And enveloped viruses with furin cleavage sites are more sensitive to ML inhibition (10). PEDV has a high risk of producing a furin cleavage site in the spike protein, so ML may be an ideal candidate to cope with this problem (24). In addition, the latest research showed that ML is a potent anti-SARS-CoV-2 active compound according to a ligand-based approach (25). Together with this study, it is shown that ML could function as a pan-coronavirus inhibitor. Further studies are warranted to confirm this hypothesis and characterize its mechanism of action.

Interferons are well-known cytokines that are produced upon virus infection. They are considered to be the first line of defense in the body (26). Interferons could specifically bind to their own receptors to stimulate the expression of ISGs, which encode direct antiviral effectors or molecules. In the early stage of disease, due to the antagonism of a small amount of virus and virus-mediated antiviral responses in the body, the expression levels of interferon are occasionally low (27). With the increase of virus replication, interferons and ISGs are dramatically induced, accompanied by the potential to cause a cytokine storm (28). Therefore, the interferon production seems to be closely associated with virus load. This hypothesis is consistent with the finding of the present study. Our results showed that ML intervention reduced the viral load in the small intestine. Coincidentally, the expression of ISGs in the ML+PEDV group was down-regulated by western blot analysis. In addition, DEPs between the PEDV group and the ML+PEDV group were significantly enriched in interferon-related pathways. This indicated that ML could promote the restoration of homeostasis by regulating the interferon pathway related to its antiviral activity.

## Conclusions

In summary, ML could alleviate diarrhea and improve intestinal function by promoting the recovery of intestinal villi. ML possessed anti-PEDV and anti-inflammatory effects by inhibiting PEDV replication and reducing the expression of IL-6 and IL-8 induced by PEDV infection, respectively. Proteomics analysis suggests ML could promote the restoration of homeostasis by regulating the interferon pathway.

## Data Availability Statement

The original contributions presented in the study are publicly available. This data can be found here: ProteomeXchange Consortium *via* the PRIDE partner repository with the dataset identifier PXD029561.

## Ethics Statement

All procedures were approved by the Animal Care and Use Committee of Wuhan Polytechnic University.

## Author Contributions

Conceptualization: YH. Methodology: QZ, DY, and CJ. Investigation: QZ, DY, CJ, TW, and MW. Data curation: DZ and LW. Writing – original draft: QZ, DY, and CJ. Writing –review and editing: QZ, DY, SG, and YH. Funding acquisition: DY and YH. Supervision: YH. All authors contributed to the article and approved the submitted version.

## Funding

This work was jointly supported by National Natural Science Foundation of China (32072762), Hubei Provincial Key R&D Program (2019ABA083), National Key R&D Program of China (2016YFD0501210), and the Optical Valley Science and Technology Innovation Corridor Project (2021BGE028).

## Conflict of Interest

The authors declare that the research was conducted in the absence of any commercial or financial relationships that could be construed as a potential conflict of interest.

## Publisher’s Note

All claims expressed in this article are solely those of the authors and do not necessarily represent those of their affiliated organizations, or those of the publisher, the editors and the reviewers. Any product that may be evaluated in this article, or claim that may be made by its manufacturer, is not guaranteed or endorsed by the publisher.
